# Morphophysiological Responses of Two Riparian Species Exposed to Water Restriction and Light Protection Conditions

**DOI:** 10.3390/plants15020259

**Published:** 2026-01-14

**Authors:** Karen Peña-Rojas, Sergio Donoso, Patricio Valenzuela-Celis, Miguel Quintanilla, Alejandro Riquelme, Claudia Espinoza, Rodrigo Gangas, Cristian Araya-Boza, Carolain Badaracco

**Affiliations:** 1 Mediterranean Forests Laboratory, Faculty of Forestry and Nature Conservation, University of Chile, Avenue Santa Rosa 11315, La Pintana 8820808, Santiago, Chile; kpena@uchile.cl (K.P.-R.);; 2 Faculty of Education, Alberto Hurtado University, Avenue Libertador Bernardo O’Higgins 1825, Santiago 9160000, Chile

**Keywords:** water restriction, light protection effects, shade-tolerant species, drought-avoidance species

## Abstract

Climate change has intensified summer drought and high solar radiation in Mediterranean ecosystems, generating abiotic stress that limits the establishment of riparian species. We conducted a nursery experiment to evaluate the effects of two levels of water availability and light intensity on the growth and physiological responses of two native riparian species from Mediterranean Chile: *Drimys winteri* and *Persea lingue*. A bi-factorial design combined two irrigation treatments (well-watered and water restriction) and two light intensity levels manipulated through a light protection treatment (20% shade mesh and full light exposure). Water restriction was applied gradually until 15–20% (*v*/*v*) substrate moisture, defined as maximum water restriction, followed by rehydration. Morphological variables (height, root collar diameter, and shoot-to-root ratio) and physiological traits (predawn water potential, chlorophyll fluorescence, and electron transport rate) were measured. Growth responses were affected by the light protection treatment, which promoted a significant height growth in both species. Water stress affected the global response of both species but they differed in their post-stress hydraulic recovery: *P. lingue* fully recovered its predawn water potential, whereas *Drimys winteri* did not. Our study provides measurable and quantifiable values that demonstrate the sensitivity of these species to water stress.

## 1. Introduction

The Mediterranean-type ecosystems are located along the western margin of South We removed the “Santiago”. Please confirm this revision.

America, extending from 30° to 36° S in central Chile [1]. These ecosystems are characterized by the transition between the Atacama Desert and mixed deciduous–evergreen temperate forest, with annual precipitation of less than 200 to 700 mm. In these regions, diverse dry xerophytic vegetation and mesic communities are characterized by the presence of evergreen sclerophyllous trees in coastal and Andean foothills, as well as by winter-deciduous trees in the southern of the Chilean Mediterranean area [2]. The “Mediterranean matorral” is notable for its patchy structure, exhibiting open spaces between shrub clumps and trees. However, in the vicinity of deep creeks, along riparian zones, and on south-facing slopes, it may evolve into a community of plants that are dependent on constant moisture (e.g., riparian zones), comprising species such as *Beilschmiedia miersii* (Gay) Kosterm, *Drimys winteri* J. R Forst. & G. Forst, *Luma chequen* A. Gray, *Citronella mucronata* (Ruiz & Pav) D. Don and *Persea lingue* Ruiz & Pav) Nees [3].

The impact of climate change on Mediterranean ecosystems is substantial, exacerbating the vulnerability of these environments due to the historical land use practices, recurrent dry years, and frequent wildfires [4,5,6,7]. Since 2010, this phenomenon has intensified under the neutral Southern Oscillation (ENSO) conditions, marked by an uninterrupted sequence of dry years observed in central Chile, resulting in annual rainfall deficits of 25–45% [8,9].

In this context, tree seedlings exhibit sensitivity to abiotic stress factors such as extreme temperatures, high radiation levels, or drought [10,11]. These stress factors are prevalent in various Mediterranean forest ecosystems. Water stress during summer drought is the primary cause of mortality among native seedlings and saplings, whether naturally regenerated or planted [12,13,14]. The seedlings belong to the hygrophyte community, are particularly vulnerable to water, light, and thermal stress, especially from midsummer to the final growing season [15,16]. In this scenario, seedlings are affected by photoinhibition and other physiological stresses induced by high temperatures and radiation during summer in the Mediterranean ecosystem [16,17,18], and therefore it is necessary to understand the role of shade tolerance, functional plasticity to variations in light intensity, and the imbalance in the plant growth of riparian species [19,20,21].

Nowadays, in Mediterranean and semiarid regions, small changes in precipitation can bring much uncertainty to the different scenarios projected for seasonal rainfall through the years [22,23]. Despite their importance and degradation, necessary research has not been conducted in riparian areas [24,25], and therefore the morphophysiological responses of riparian vegetation have not been entirely quantified when declining the stream base flows [26].

Some experiments suggest a positive effect of tree “shelters” and mesh on the abiotic stress incidence of shade-semi-tolerant species [27,28]. Other studies show that light reduction within the tree shelter limits the survival of shade-intolerant species [29,30,31], with responses such as an increase in leaf-specific area, stomatal density change and biomass allocation in shoots, and a reduction in root growth [32]. Therefore, beneficial alleviation depends on the species’ characteristics and environmental conditions, with shade-intolerant species not benefiting from light shelter as much as shade-tolerant species [33]. Knowing how riparian species face water restriction periods through Chilean Mediterranean ecosystems is crucial due to their susceptibility to water stress [34,35].

An improvement in the adequacy of the plantation will be critical to ensure subsequent success and thus avoid the stress associated with a water deficit along with the increase in irradiation in the summer [36]. Likewise, the light transmissivity reduction in seedlings affected by water stress can reduce their photoinhibition, but more information is still necessary for many species [26,37]. There need to be more studies on how this water and light restriction combination would affect seedling performance during initial establishment, considering that it is crucial to know about the effect of drought and shadow on riparian species in climate change conditions. To this end, there must be increased knowledge about morphophysiological responses in the variability of those ecosystems to improve the site management of water deficit and light excess during plant establishment.

A nursery study was conducted to evaluate the effects of two different levels of water availability and light intensity on plant growth and physiological responses during early establishment. The objectives of the study were to evaluate the effects of water restriction and light protection on two-year-old plants of two riparian species and semi-shade-tolerant species such as *Drimys winteri* and *Persea lingue*. It was expected that *Drimys winteri* would show greater water and light stress compared to *Persea lingue*.

## 2. Methods

### 2.1. Study Site

The experimental site was in Santiago de Chile, in Antumapu nursery of the University of Chile, Faculty of Forest Sciences and Nature Conservation (FCFCN), located in La Pintana district (70°38′ W and 33°34′ S, elevation 605 masl). The zone has a warm temperate suprathermal climate with a semiarid humidity regime and annual temperature of 13.9 °C. During the summer, the mean temperature is 22.1 °C (peak above 31 °C) and, in winter, the mean reaches 7.7 °C. The mean annual rainfall is 371 mm, with a dry period of 8 months and a water deficit of 1150 mm/year. The wet period is three months (May to July), in which a water surplus of 48 mm is produced [38].

### 2.2. Experimental Design

This study used 80 two-year-old *D. winteri* plants with similar development, height, and health status. We brought plants from the “Las Vertientes” nursery, located in the Paine district (50 km to the study site). All plants came in plastic containers of 9500 cm^3^ (20 × 30 cm). We also brought 108 two-year-old *P. lingue* plants from “Cauquenes” plant nursery in Requinoa district (104 km to the study site). These came in plastic bags of 4500 cm^3^ (15 × 25 cm). Both plant groups had similar morphological characteristics, good development of lateral branches, and good phytosanitary condition. All plants were transplanted into pots (11,000 cm^3^). The pots were filled with a mixture of local soil (Santiago Serie, Entic Haploxerolls (Mollisol)) and organic matter (litter from riparian forests) in a 2:1 ratio. Then, for one month, all the plants were grown in field capacity conditions, as a way to homogenize the initial conditions of the plants before the trial. For both species, we evaluated a bi-factorial model with two levels for each factor: (1) water availability (well-watered and water-restricted) and (2) light intensity (light-protected and not protected).

The light protection treatment considered the use of green raschel mesh with a 20% light transmissivity mesh, and it was implemented around each individual plant; the mesh was 120 cm tall × 25 cm wide.

The water restriction protocol was implemented following an established methodology [39]. Substrate moisture was gradually reduced to 15–20% water content (*v*/*v*) over 90 days from the beginning of the study, representing the maximum level of water restriction prior to plant mortality. After this stage, all the plants reached field capacity until the end of the study.

**Figure 1:**
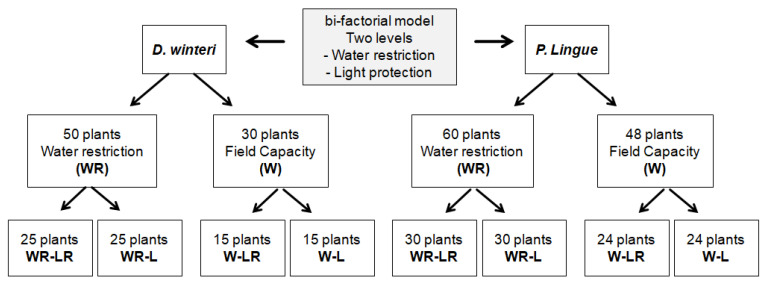
Scheme of the bi-factorial model in the study with *D. winteri* and *P. lingue* exposed to different levels of water availability and light intensity. For both species, treatments were as follows: (a) water restriction and light protection (WR-LR); (b) water restriction and no light protection (WR-L); (c) field capacity and light protection (W-LR), and (d) no light protection (W-L).

A total of 80 *D. winteri* plants were utilized, with 50 plants subjected to water restriction; 25 had light protection (WR-LR), and 25 had no light protection (WR-L). Additionally, 30 plants were subjected to well-watering conditions, defined as field capacity; 15 of these plants received light protection (W-LR) and 15 plants received no light protection (W-L) (Figure 1).

In the case of *P. lingue*, 108 plants were utilized, of which 60 plants were exposed to water restriction; of these, 30 plants had light protection (WR-L) and 30 plants had no light protection (WR-L). Additionally, 48 plants were subjected to well-watering conditions (field capacity); 24 plants were provided with light protection (W-LR), while 24 plants had no light protection (W-L) (Figure 1).

Water restriction was carried out in three stages: (1) initial water restriction; (2) maximum water restriction; (3) the rehydration stage.

For *D. winteri*, the water restriction began on 12 January 2018 and ended on 5 June 2018. For *P. lingue*, water restriction began on 31 January 2019 and ended on 5 June 2019.

### 2.3. Plant Measurement

#### 2.3.1. Monitoring Plant Responses During the Study

Initially, we randomly selected fifteen *D. winteri* plants per treatment and monitored the root collar diameter (RCD) and height growth throughout the study. For *P. lingue*, we chose ten plants per treatment and we conducted repeated measures of RCD and height growth.

For the physiological measurements, we randomly selected six plants per treatment and evaluations took place every 15 days for both species. These evaluations included information about water potential, photosynthetic responses through chlorophyll fluorescence, and height and root collar diameter. This information was obtained at the beginning of the study, at maximum water restriction, and at end of the rehydration stage. The registers obtained were predawn leaf water potential (Ψpd); the maximum photochemical efficiency of photosystem II at predawn (Fv/Fmpd); photosystem II photochemical efficiency (ΦPSII); photochemical quantum yield (PQ); non-photochemical quantum yield (NQP); and the electron transport rate (ETR).

We evaluated water stress and fluorescence condition with a random leaf sample from each of the selected plants, considering healthy leaves and an appropriate petiole length. For water potential (Ψpd), we used a Plant Moisture Stress Pressure Chamber (PSI System Model 1000, PMS Instrument Company, Albany, OR, USA). For fluorescence, we measured fully developed leaves with a portable fluorometer (Mini-PAM Photosynthesis Yield Analyser. Walz, Effeltrich, Germany).

At the end of the rehydration phase, we randomly selected four plants per treatment, destroyed them (roots, stems, and leaves), and measured the shoot-to-root ratio. They were dried in an oven at 65 °C until they reached a stable weight, at which point plant biomass was newly measured and the shoot-to-root ratio was calculated again.

#### 2.3.2. Statistical Analysis During the Study

The information was collected during June 2018 (*D. winteri*) and June 2019 (*P. lingue*), and multivariate analysis of variance (MANOVA) was assessed to test the statistical significance of the effect of the two factors (water availability and light intensity) on height, RCD, Ψpd, Fv/Fmpd, FPSII, PQ, NPQ, and ETR. After that, we performed two-factor analysis of variance (ANOVA) for every dependent variable, considering every individual dependent variable at the beginning of water restriction, at the point of maximum water restriction and after the rehydration phase.

Through these assessments, it was possible to contrast global and individual effects from factors on dependent variables. A logarithmic transformation was made to fulfill normality and variance homoscedasticity requirements for non-normal data. We performed a post hoc Tukey test with a confidence level of 95% to demonstrate statistical significance among means. All analyses were performed with R software 2022 version 4.2.0 (The R Foundation for Statistical Computing).

## 3. Results

### 3.1. MANOVA Analysis Results

We found that water availability and light intensity affected the global morphophysiological response of *D. winteri* and *P. lingue* differently.

The water restriction factor influenced the global response of *D. winteri* at maximum water restriction. This factor was still significant after the rehydration stage. It was at this point when the interaction of the factors also appeared as a significant influence on the global response of the species (Table 1a; *p* < 0.05).

In *P. lingue*, water restriction was also significant at maximum water restriction but this effect disappeared after the rehydration process. The effect of light protection was only significant at the end of the study after the rehydration process (Table 1b; *p* < 0.05).

### 3.2. Plant Growth Results

To examine the effects of the factors on growth, we measured root collar diameter growth (RCD) and height growth (HG) at the beginning of the study, at the point of maximum water restriction, and during the rehydration stage.

**Figure 2:**
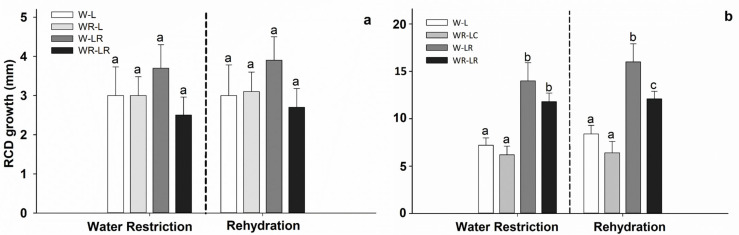
The root collar diameter (RCD; (**a**)), and height growth (HG; (**b**)) for *D. winteri* from the beginning to the end of water restriction and the beginning of the rehydration of the plants. The treatments were (a) water restriction and light protection (WR-LR); (b) water restriction and no light protection (WR-L); (c) field capacity and light protection (W-LR); and (d) no light protection (W-L). Bars represent mean values ± standard error. No significant differences were observed among the mean for RCD. For height growth, different letters in bars denote differences among treatments. *p* < 0.05.

In *D. winteri*, water restriction and light protection did not have a significant effect on RCD, with an increase between 2.7 mm and 3.9 mm but extended standard deviation (Figure 2). Both treatments significantly affected the HG when the beginning of the study was compared with the rehydration stage. Only light protection treatment had a significant effect on HG when we compared the beginning of the study with the maximum water restriction (Table 2; Figure 2a), (Figure 2b; *p* < 0.05). At the end of the study, the tallest saplings were those from W-LR treatment (16.0 ± 1.9 cm) and the smallest were those from WR-L treatment (6.4 ± 1.2 cm).

**Figure 3:**
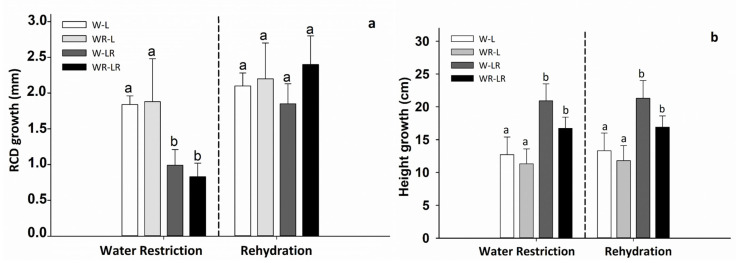
The root collar diameter (RCD; (**a**)) and height growth (HG; (**b**)) for *P. lingue* from the beginning to the end of water restriction and the beginning of the rehydration of the plants. The treatments were (a) water restriction and light protection (WR-LR); (b) water restriction and no light protection (WR-L); (c) field capacity and light protection (W-LR); and (d) no light protection (W-L). Bars represent mean values ± standard error. No significant differences among the means were observed in RCD. For height growth, the different letters in the bars denote differences among treatments after Tukey’s post hoc test. *p* < 0.05.

For *P. lingue*, the light protection showed significant differences in the RCD and HG at beginning of the study compared with those reported at the point of maximum water restriction. In HG, this difference was also maintained during the rehydration stage (Table 2; *p* < 0.05), where plants with light restriction had a HG averaging between 16 and 21 cm (Figure 3b; *p* < 0.05).

**Figure 4:**
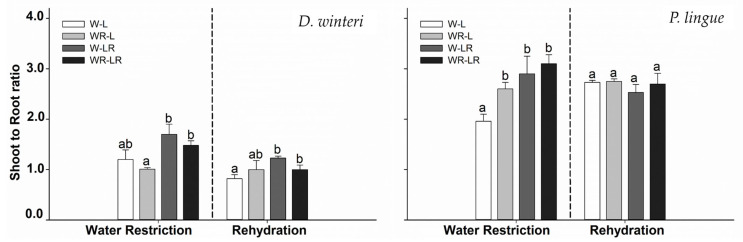
The shoot-to-root ratio for *D. winteri* and *P. lingue* from the beginning to the end of water restriction and the beginning of the rehydration of the plants. The treatments were (a) water restriction and light protection (WR-LR); (b) water restriction and no light protection (WR-L); (c) field capacity and light protection (W-LR); and (d) no light protection (W-L). Bars represent mean values ± standard error. No significant differences among the means were observed in RCD. For height growth, different letters in bars denote differences among treatments after Tukey’s post hoc test. *p* < 0.05.

The biomass growth showed a significant effect on the shoot-to-root ratio of *D. winteri* (*p* < 0.05), showcasing the principal differences between plants from W-LR (1.26 ± 0.04) and W-L (0.80 ± 0.08) treatments at the end of rehydration (Table 3; Figure 4). On the other hand, stem biomass, leaf biomass, and root biomass registers were not statistically significant (Table 3). *P. lingue* had significant differences among treatments for the shoot-to-root ratio after water restriction, but this difference disappeared at the end of the rehydration process (Figure 4).

### 3.3. Physiology Results

The hydric response of *D. winteri* showed that the predawn water potential (Ψpd) was affected by the water restriction factor at the maximum water restriction stage. It was during this stage that we registered the lowest values of mean Ψpd between all treatments, specifically in the WR-L treatment (−1.1 ± 0.16 MPa). At this Ψpd, we observed signals of critical damage, such as apical death. With the rehydration phase, the plants could not recover their initial condition in terms of this parameter (Table 4; *p* < 0.05).

The hydric response of *P. lingue* showed that the water restriction factor affected the predawn water potential (Ψpd) at the maximum water restriction stage, but the plants recovered their initial condition with rehydration (Table 5; *p* < 0.05). Among treatments, plants exposed to water restriction and light protection (WR-LR) demonstrated the lowest values of mean Ψpd (−1.40 ± 0.30 MPa) at maximum water restriction. At this Ψpd, we observed signs of critical damage, such as defoliation. At the end of rehydration, the value of the mean Ψpd was higher for this treatment, reaching −0.42 ± 0.08 MPa.

The photosynthetic response of *D. winteri* evidenced that PSII, NPQ, and ETR were significantly influenced by the light protection at maximum water restriction, but this effect did not appear at the end of the rehydration stage (Table 4 *p* < 0.05). The maximum fluorescence ratio (Fv/Fm) at predawn did not show any significant differences between stages (Table 4 *p* < 0.05). The photosynthetic response of *P. lingue* did not show any significant difference between the initial condition of photosynthetic parameters and the values at the maximum water restriction stage (Table 5 *p* < 0.05). However, significant differences in some photosynthetic parameters between the initial and rehydration stage were evident due to the effect of light protection factor (Table 5; *p* < 0.05; PSII and PQ parameters).

## 4. Discussion

Our study provides novel ecophysiological insights into the differential vulnerability of two native riparian species, *Drimys winteri* and *Persea lingue*, to combined water and light stress in the context of intensifying Mediterranean droughts. While the parameters measured (predawn water potential, chlorophyll fluorescence, and growth traits) are well-established, their integration in a factorial experiment combining water restriction, light modulation, and post-stress rehydration, applied to co-occurring hygrophilous species from a climate-vulnerable biome, reveals previously undocumented contrasts in functional strategy and recovery capacity.

### 4.1. Divergent Hydraulic Recovery: Evidence of Contrasting Drought-Coping Syndromes

A central finding is the species-specific capacity for hydraulic recovery after rehydration. *Persea lingue* fully restored its predawn water potential (Ψpd), suggesting either minimal xylem embolism or efficient refilling mechanisms, a hallmark of drought-resilient species [40,41]. In contrast, *Drimys winteri* exhibited irreversible Ψpd decline, indicative of hydraulic failure, consistent with its limited stomatal regulation and strong coupling between soil moisture and sap flux [42,43].

Although predawn water potential is a widely used proxy for plant water status, it does not directly quantify xylem embolism or hydraulic conductivity loss. Full recovery of Ψpd, as observed in *P. lingue*, suggests effective rehydration of living tissues and possibly minimal embolism or efficient refilling mechanisms. In contrast, the failure of *D. winteri* to recover Ψpd despite rehydration implies irreversible hydraulic impairment, even though stress levels (−1.1 to −1.4 MPa) remained above typical embolism thresholds. This highlights that species-specific vulnerabilities, such as limited parenchyma-mediated refilling or high stomatal–sapwood coupling, can lead to functional hydraulic failure at relatively moderate water potentials [40,41,43].

This divergence is ecologically significant: it positions *P. lingue* as a resilient riparian engineer capable of withstanding intermittent drought, while *D. winteri* functions as a hydrological sentinel, highly sensitive to even moderate declines in soil water availability.

This contrast transcends a simple stress response description; it reveals two distinct drought-coping syndromes within the same ecological niche: one oriented toward hydraulic resilience (*P. lingue*), and another toward hydrological fidelity (*D. winteri*). Such functional differentiation among co-occurring riparian taxa has rarely been experimentally demonstrated in Mediterranean ecosystems, particularly under post-stress recovery scenarios [33,40].

### 4.2. Photoprotection Enhances Growth Even Under Water Deficit: Implications for Restoration

Both species exhibited significantly greater height growth under 20% shade, even during water restriction, a finding with direct practical relevance. This challenges the assumption that light reduction benefits seedlings only under well-watered conditions. Instead, our results support the hypothesis that moderate shading mitigates the synergistic stress of high irradiance and drought, particularly for semi-shade-tolerant species with limited non-photochemical quenching (NPQ) capacity [44,45].

For *D. winteri*, light protection also increased the shoot-to-root ratio, reflecting a classic shade-avoidance response [32]. However, in *P. lingue*, the growth benefit under shade persisted through rehydration, suggesting that light modulation not only reduces photoinhibition during stress but also enhances post-drought recovery potential. This provides strong empirical support for the use of 20% transmissivity mesh shelters in riparian restoration nurseries and field plantings, a low-cost, scalable intervention to improve seedling establishment under climate uncertainty [37].

The growth enhancement under 20% shade, even during water restriction, is likely mediated by two interconnected mechanisms. First, reduced photon flux density lowers the excitation pressure on photosystem II (PSII), decreasing the generation of reactive oxygen species (ROS) and minimizing photoinhibitory damage during periods of stomatal closure [45]. Second, moderate shading reduces leaf temperature and vapor pressure deficit (VPD), which in turn lowers transpirational demand and helps maintain higher leaf water potentials, which are particularly critical for hygrophilous species with limited stomatal control [33,44]. In *P. lingue*, this dual benefit translated into sustained height growth through rehydration, suggesting that light modulation not only mitigates stress during drought but also preserves recovery capacity afterward.

### 4.3. Riparian Species as Sentinels of Hydrological Change

Critically, both species exhibited stress and damage thresholds at Ψpd values markedly less negative (−1.1 to −1.4 MPa) than those reported for co-occurring Mediterranean sclerophytes such as *Quillaja saponaria* (−3.0 MPa) [13] or *Cryptocarya alba* (−2.5 MPa) [46]. This confirms their exceptional sensitivity to water availability, a trait expected for species that have adapted to stable riparian moisture regimes. In a region experiencing a decade-long mega-drought [8,9], such sensitivity makes *D. winteri* and *P. lingue* early-warning bioindicators of declining stream base flows and altered hydrological connectivity.

This applied insight, using physiological thresholds to identify sentinel species for ecosystem monitoring, represents a conceptual advance over descriptive stress-response studies. It aligns with emerging frameworks for climate-resilient conservation that prioritize functional vulnerability over taxonomic presence alone [25,26,33].

### 4.4. Limitations and Future Directions

We acknowledge that the experiments were conducted in consecutive years (2018–2019), though under highly similar climatic conditions during Chile’s ongoing mega-drought. While within-species comparisons remain robust, future work should replicate this design simultaneously for both species to enable direct interspecific statistical contrasts. Additionally, linking these physiological responses to field survival and growth would strengthen the applied recommendations.

## 5. Conclusions

Our study moves beyond the description of stress responses by revealing functional divergence in post-drought hydraulic recovery, demonstrating the photoprotective value of moderate light reduction under water deficit, and proposing native riparian species as early-warning bioindicators of hydrological change.

*Drimys winteri* exhibited irreversible hydraulic damage under water restriction, failing to recover its predawn water potential after rehydration, which confirms its role as a hydrological sentinel that is highly sensitive to soil moisture decline. In contrast, *Persea lingue* fully restored its water status, highlighting its hydraulic resilience and potential suitability for restoration in increasingly arid riparian zones.

Furthermore, 20% light protection significantly enhanced height growth in both species (even under water stress) supporting its use as a low-cost, scalable intervention in nursery and outplanting protocols.

Given their narrow hydraulic safety margins compared to co-occurring Mediterranean sclerophytes, both species are more vulnerable to summer drought than previously assumed. This underscores their ecological value not as generalist trees, but as sensitive indicators of ecosystem water balance in a drying world.

Altogether, these findings fill critical knowledge gaps in Mediterranean riparian ecology and provide actionable insights for climate-adaptive forest restoration.

## Figures and Tables

**Table 1:** Multivariate analysis of variance (MANOVA) for the water and light restriction effects on physiological responses from *D. winteri* (**a**) and *P. lingue* (**b**). This analysis considered results at each time of evaluation and took into account the predawn leaf water potential (Ψpd); variable and maximum fluorescence ratio at predawn (Fv/Fmpd); Photosystem II photochemical efficiency openness (PSII); photochemical quantum yield (PQ); non-photochemical quantum yield (NPQ); and the electron transport rate (ETR). Logarithmic transformation was performed when normality and homoscedasticity were not found (significance *p* < 0.05). The dataset underlying these results is provided in Supplementary Materials Table S1.

(a)					
*D. winteri*	Factor	DF	Pillai Index	F Value Aprox	*p*-Value
Beginning	Water restriction	1	0.290	0.740	0.629
	Light protection	1	0.270	0.680	0.669
	Interaction	1	0.540	2.130	0.131
Maximum Water Restriction	Water restriction	1	0.580	3.430	0.025
	Light protection	1	0.470	2.210	0.100
	Interaction	1	0.230	0.740	0.626
End of Rehydration	Water restriction	1	0.580	3.520	0.022
	Light protection	1	0.360	1.390	0.28
	Interaction	1	0.700	5.700	0.003
**(b)**					
***P. lingue***	**Factor**	**DF**	**Pillai Index**	**F Value Approx.**	***p*** **-Value**
Beginning	Water restriction	1	0.396	1.091	0.429
	Light protection	1	0.550	2.037	0.153
	Interaction	1	0.059	0.105	0.994
Maximum Water Restriction	Water restriction	1	0.750	7.320	0.001
	Light protection	1	0.440	1.960	0.136
	Interaction	1	0.360	1.400	0.278
End of Rehydration	Water restriction	1	0.170	0.530	0.779
	Light protection	1	0.640	4.440	0.010
	Interaction	1	0.500	2.510	0.069

**Table 2:** The ANOVA results of growth responses of *D. winteri* and *P. lingue* exposed to water restriction and light protection during the study. The variables evaluated were root collar diameter growth (RCD) and height growth (HG). Logarithmic transformation was performed when normality and homoscedasticity were not found (significance *p* < 0.05).

Factors	*D. winteri*				*P. lingue*			
	RCD		HG		RCD		HG	
	F	*p*-Value	F	*p*-Value	F	*p*-Value	F	*p*-Value
Beginning–Maximum Water Restriction								
Water restriction	1.05	0.31	2.7	0.10	0.90	0.34	0.43	0.51
Light protection	0.0001	0.99	24.30	<0.01	11.10	<0.01	8.4	<0.01
Interaction	1.22	0.27	0.15	0.70	0.02	0.87	0.06	0.79
Beginning–Rehydration								
Water restriction	1.04	0.31	5.1	0.02	0.10	0.74	0.61	0.43
Light protection	0.06	0.80	19.80	<0.01	0.11	0.73	7.6	<0.01
Interaction	1.69	0.19	0.54	0.46	0.97	0.33	0.04	0.84

**Table 3:** ANOVA results corresponding to biomass measurements of *D. winteri* and *P. lingue* exposed to water restriction and light protection during the study. The variables evaluated were leaf biomass (LB), stem biomass (SW), root biomass (RB), and shoot-to-root ratio (S/R). In this analysis, we only considered the result after rehydration, and logarithmic transformation was performed when normality and homoscedasticity were not found (significance *p* < 0.05).

Variables Beginning–Rehydration	Stem Biomass (SB)		Leaf Biomass (LB)		Root Biomass (RB)		Shoot-to-Root Ratio	
	F	*p*-Value	F	*p*-Value	F	*p*-Value	F	*p*-Value
Drimys winteri								
Water restriction	0.0001	0.99	0.01	0.91	0.03	0.86	0.29	0.58
Light protection	0.07	0.78	1.24	0.27	0.04	0.83	4.80	0.03
Interaction	0.09	0.75	0.06	0.80	0.004	0.94	0.46	0.49
Persea lingue								
Water restriction	0.57	0.45	2.03	0.16	0.16	0.69	1.02	0.32
Light protection	3.30	0.08	0.18	0.66	1.85	0.18	1.39	0.24
Interaction	0.01	0.90	0.10	0.74	0.39	0.53	0.29	0.58

**Table 4:** ANOVA evaluation of physiological responses from *D. winteri* exposed to water and light restriction during the study. The variables evaluated were predawn leaf water potential (Ψpd); variable and maximum fluorescence ratio at predawn (Fv/Fmpd); Photosystem II photochemical efficiency openness (ΦPSII); photochemical quantum yield (PQ); non-photochemical quantum yield (NPQ); and the electron transport rate (ETR). Logarithmic transformation was performed when normality and homoscedasticity were not found (significance *p* < 0.05). The dataset underlying these results is provided in Supplementary Materials Table S1.

*D. winteri*	Ψ_pd_				Fv/Fm_pd_			
	F		*p*-Value		F		*p*-Value	
Beginning–Maximum Water Restriction								
Water restriction	59.61		<0.01		1.45		0.25	
Light protection	13.69		<0.01		4.29		0.06	
Interaction	4.30		0.06		0.13		0.73	
Beginning–Rehydration								
Water restriction	22.19		<0.01		2.89		0.11	
Light protection	0.41		0.53		1.04		0.32	
Interaction	0.09		0.77		0.25		0.63	
***D. winteri***	**ΦPSII**		**PQ**		**NPQ**		**ETR**	
	**F**	***p*-Value**	**F**	***p*-Value**	**F**	***p*-Value**	**F**	***p*-Value**
Beginning–Maximum Water Restriction								
Water restriction	1.94	0.18	0.13	0.72	6.29	0.02	2.49	0.14
Light protection	7.54	0.02	2.01	0.18	12.51	<0.01	8.77	0.01
Interaction	0.31	0.58	0.01	0.94	2.29	0.15	1.89	0.19
Beginning–Rehydration								
Water restriction	0.02	0.90	2.30	0.15	1.27	0.28	2.05	0.17
Light protection	1.06	0.32	0.24	0.63	0.57	0.46	1.92	0.19
Interaction	0.81	0.38	12.85	<0.01	1.34	0.27	0.37	0.55

**Table 5:** ANOVA evaluation of physiological responses from *P. lingue* exposed to water and light restriction during the study. The variables evaluated were predawn leaf water potential (Ψpd); variable and maximum fluorescence ratio at predawn (Fv/Fmpd); Photosystem II photochemical efficiency openness (ΦPSII); photochemical quantum yield (PQ); non-photochemical quantum yield (NPQ); and the electron transport rate (ETR). Logarithmic transformation was performed when normality and homoscedasticity were not found (significance *p* < 0.05). The dataset underlying these results is provided in Supplementary Materials Table S1.

*P. lingue*	Ψ_pd_				Fv/Fm_pd_			
	F		*p*-Value		F		*p*-Value	
Beginning–Maximum Water Restriction								
Water restriction	31.45		<0.01		0.36		0.56	
Light protection	0.26		0.62		7.31		0.02	
Interaction	1.95		0.18		3.22		0.09	
Beginning–Rehydration								
Water restriction	0.45		0.51		0.34		0.57	
Light protection	11.79		<0.01		9.49		0.01	
Interaction	1.64		0.22		0.06		0.81	
***P. lingue***	**ΦPSII**		**PQ**		**NPQ**		**ETR**	
	**F**	***p*-Value**	**F**	***p*-Value**	**F**	***p*-Value**	**F**	***p*-Value**
Beginning–Maximum Water Restriction								
Water restriction	0.06	0.81	0.07	0.80	0.10	0.76	0.05	0.82
Light protection	0.010	0.93	0.12	0.73	1.35	0.27	0.01	0.94
Interaction	0.17	0.69	0.07	0.80	0.07	0.79	0.17	0.69
Beginning–Rehydration								
Water restriction	0.25	0.63	2.39	0.14	1.27	0.28	3.34	0.09
Light protection	3.79	0.07	3.99	0.07	0.26	0.62	1.20	0.29
Interaction	0.90	0.36	7.26	0.02	0.24	0.63	4.29	0.06

## Data Availability

The dataset supporting this study are included in the Supplementary Material. Further inquiries can contact the corresponding author.

## Supplementary Materials

The following supporting information can be downloaded at: https://www.mdpi.com/article/10.3390/plants15020259/s1, Table S1: Original dataset.
